# The availability between recreational facilities and physical activity of US adolescents

**DOI:** 10.1016/j.pmedr.2024.102592

**Published:** 2024-01-05

**Authors:** Jamee Guerra, Johnatan Jhon, Kevin Lanza, Grettel Castro, Noël C. Barengo

**Affiliations:** a Facultad de Ciencias de la Salud, Universidad Privada del Norte, Lima, Perú; b Department of Medical Education, Herbert Wertheim College of Medicine, Florida International University, Miami, FL, USA; c University of Texas Health Science Center at Houston School of Public Health, Austin, TX, USA; d Escuela Superior de Medicina, Universidad Nacional de Mar del Plata, Mar del Plata, Argentina

**Keywords:** Adolescents, Physical activity, Recreational facilities, Environment, Exercise

## Abstract

Determining the locations where adolescents tend to accumulate greater amounts of physical activity may assist policymakers to address the built environment design and promote PA. This study evaluated the association between the availability of recreational facilities and average minutes of moderate to vigorous physical activity (MVPA) per day of US adolescents in 2017 (n = 1,437). Data for this cross-sectional study were obtained from the 2017 Family Life, Activity, Sun, Health, and Eating study, an internet-based study collecting information on diet and PA of parent and adolescent dyads. Adolescents aged 12–17 from the US were included. Predicted daily minutes of MVPA were calculated. The exposure variables of interest were the availability of school recreational facilities, indoor recreational facilities, playing fields, bike/hiking/walking trails or paths or public parks. Participants were excluded if no information was provided for MVPA or availability of recreational facilities. Unadjusted and adjusted linear regression analysis was used to calculate mean daily minutes of MVPA and their corresponding 95 % confidence intervals. In fully adjusted models, we found statistically significant associations between the type of recreational facility and differences in daily minutes of MVPA for school (p-value < 0.001) and public parks p-value < 0.001), but not for the other recreational facilities. The average daily minutes of MVPA differed by 4.4 min (95 % CI 2.6, 6.2) if participants had school recreational facilities, respectively. School recreational facilities are important for engaging adolescents in PA objectives. Features within school recreational facilities should be studied to further investigate contributions to increased PA levels.

## Highlights

• Children and adolescent school recreational facilities availability out of school hours were only present in less than half of the US schools.
• School recreational facilities were positively associated with moderate to vigorous physical activity.
• The average daily minutes of MVPA differed by 4.4 min (95 % CI 2.6, 6.2) if participants had school recreational facilities.
• Physical activity with friends and disagreeing with family physical inactivity status was also associated with daily minutes of moderate to vigorous physical activity.

## 1 Introduction

The health benefits of physical activity (PA) are well documented (World Health Organization. WHO Guidelines on physical activity and sedentary behaviour., 2020, Wyszyńska et al., 2020). Regular physical activity is important for children and adolescents since it decreases the risk of future cardio-metabolic diseases (Janssen et al., 2005). Further, adolescents who are physically active are more likely to engage in PA in adulthood (Jones et al., 2013). Current PA recommendations from World Health Organization (WHO) and US guidelines call for adolescents to participate in an average of 60 min of moderate-to-vigorous physical activity (MVPA) daily (World Health Organization. WHO Guidelines on physical activity and sedentary behaviour., 2020, Department of Health and Human Services, 2018), yet it is globally estimated that less than 20 % of adolescents achieve this goal (Guthold et al., 2020, World Health Organization. Prevalence of Insufficient Physical Activity among adolescents aged 11-17 years, 2016), a pattern replicated in the US (World Health Organization. Prevalence of Insufficient Physical Activity among adolescents aged 11-17 years, 2016, Marques et al., 2020). Consequently, research related to increasing PA and assessing its determinants among adolescents has been of great interest (Wilk et al., 2018, Kelso et al., 2021, Thornton et al., 2017).

PA is a complex behavior influenced by individual, social, and environmental factors (Wyszyńska et al., 2020, da Silva et al., 2017). During the past two decades, the focus has been on interactions between individuals and their physical and socio-cultural environments (Sallis et al., 2006, Bauman et al., 2012). Because PA patterns of adolescents differ from those of adults (Szeszulski et al., 2021), environmental factors studied in youth populations may benefit from a specific approach as proposed in a conceptual framework model (Szeszulski et al., 2021) that accounts for the type of PA and where and when it occurs. When different environmental settings are analyzed, recreational facilities have accounted for up to 56.4 min of MVPA per day (Kelso et al., 2021). However, multiple types of recreational facilities have been merged into this single term, which in turn may under- or over-estimate their actual contribution. There is a knowledge gap about the contribution of specific recreational facilities to PA of adolescents (Szeszulski et al., 2021).

Recreational facilities, whether public or private, are considered as an area or facility constructed on a lot, parcels, or tract of land primarily for periodic and short-term sports or personal leisure activities. The availability of recreational facilities near an adolescent’s home may be of great importance since they have less autonomy in their ability to visit places located at a greater distance from their residence (Van Hecke et al., 2018, Ding et al., 2011). While some studies have reported that the availability of general recreational facilities contributes to the MVPA in the adolescent population, mixed results have been found (Kelso et al., 2021), which emphasize the need to assess the association between the availability of different recreational facilities and PA among a nationally representative sample of US adolescents. The objective of this study was to evaluate the association between the availability of recreational facilities and average minutes of moderate to vigorous physical activity (MVPA) per day of US adolescents in 2017. Determining the locations where adolescents tend to accumulate greater amounts of MVPA may assist policymakers to address the built environment design and promote positive PA behaviors that could further impact PA behavior into adulthood.

## 2 Methods

### 2.1 Study design and participants

Data for this cross-sectional study were obtained from the 2017 Family Life, Activity, Sun, Health, and Eating (FLASHE) study, an internet-based study collecting information on diet and PA of parent and adolescent dyads (Nebeling et al., 2017). FLASHE obtains its sample using an Ipsos Consumer Opinion Panel, which is representative of the United States general population based on age, sex, education, income, household size, and Census region. Once an eligible household was identified, one adolescent (ages 12–17) for the household was randomly selected to participate in the survey. Responses were collected from April to October of 2014 via internet surveys. A total of 1,945 dyads were fully enrolled in the study The current research investigation was focused on the subset of adolescent respondents ages 12–17 years who completed the FLASHE Annotated Teen Physical Activity Survey and the FLASHE Annotated Teen Demographic Survey. Participants were excluded if data were missing for either the exposure variables or outcome variables. A total of 1,682 participants ages 12–17 responded to the FLASHE physical activity and demographics survey in 2017. Of these, 58 participants did not respond to questions regarding access to recreational facility type and were therefore excluded. Additionally, participants were excluded if there were missing data for minutes of PA in school (150 participants), out of school (150 participants), and on weekends (135 participants). The final sample consisted of 1,437 US adolescents. 

### 2.2 Study variables

#### 2.2.1 Measurement of moderate-to-vigorous PA (MVPA)

The dependent variable was the average minutes of MVPA per day, which was assessed using the Youth Activity Profile (YAP). The YAP score is a quantification of participant responses to PA questions answered on a 5-point Likert Scale, with higher values indicating higher levels of MVPA (Nebeling et al., 2017). Previously, YAP scores have been validated against accelerometer data and their correlation value was higher than those observed in other large population studies using different PA measures (r = 0.52) (Saint-Maurice and Welk, 2015). The 15-item self-report instrument was designed to measure PA in a previous seven-day recall period. It is divided into sections accounting for PA behavior at school and out-of-school, with the latter further subdivided by weekday and weekend (Saint-Maurice and Welk, 2015). These two sections were utilized in the current study to estimate MVPA daily for a given week. Each YAP item was on a Likert Scale (1–5) and the average score at school, out-of-school, and on weekends was obtained and separately calibrated by the YAP score at each category and by age (Saint-Maurice et al., 2017). Once calibrated, each YAP composite was utilized to predict minutes of MVPA using regression models for at-school, out-of-school, and weekends (Saint-Maurice et al., 2017). Finally, predicted weekly minutes of MVPA at school, out-of-school, and on weekends were summed and then divided by seven to represent the average daily MVPA for a given week. In this survey, PA was defined as “any play, game, sport, exercise or transportation (like walking or biking to school) that gets you moving and breathing harder” and includes “structured exercise or sports activities as well as actively playing with friends, dancing, or doing work/chores”.

#### 2.2.2 Measurement of recreational facilities

The five independent variables of interest in this study were whether the participant had access to school recreational facilities, indoor recreational facilities, playing fields (Basketball courts, running track/other playing fields (like soccer, football, softball, tennis, skate park), Bike/hiking/walking trails/paths or public parks in their neighborhood. The following question with five possible response options was asked to determine the recreational facilities available to participants: “Please indicate if you have the following in your neighborhood: Indoor recreation or exercise facility (public or private); School with recreation facilities open to the public; Bike/hiking/walking trails, paths; Basketball courts, running track/other playing fields (like soccer, football, softball, tennis, skate park, etc.); public parks. Participants could select more than one option.

#### 2.2.3 Measurement of covariates

The analysis included several covariates—age, sex, school grade, race/ethnicity, school type, work status, perceived neighborhood crime, perceived neighborhood traffic, PA with friends, perceptions of friends’ PA, and perceptions of family physical inactivity all of which have been found to exhibit associations with MVPA (Sterdt et al., 2014, Wilkie et al., 2018). Age was treated as a continuous variable. Sex was reported as male or female. School grades were dichotomized into middle school or below and high school. Race/ethnicity was reported as either White alone, Black alone, Hispanic, or another race/ethnicity. School type was differentiated by public, private, or other. Parent work status was dichotomized as working or not. Perceived neighborhood crime and perceived neighborhood traffic were reported as either strongly agree, somewhat agree, somewhat disagree, or strongly disagree. PA with friends, perceptions of friends’ PA, and perceptions of family physical inactivity were reported as either agree, neutral or disagree.

### 2.3 Statistical analysis

All statistical analysis was completed in STATA version 16.1. First, descriptive analysis was conducted to check for missing data and construct the variables for analysis. Then, bivariate analyses were conducted to assess for possible confounders utilizing chi-square testing on categorical variables, as well as t-tests and ANOVA on continuous variables (Table 1). If the covariates exhibited statistically significant associations with the exposure and/or the outcome (p-value < 0.001), they were included in the regression model. Collinearity diagnostics were conducted to determine the extent of correlation between study variables. Finally, unadjusted and adjusted linear regression models were computed to calculate mean daily minutes of MVPA and corresponding 95 % confidence intervals.

**Table 1:** Daily minutes of moderate to vigorous physical activity in adolescents according to their characteristics in 2017.

	**Minutes of Moderate to Vigorous Physical Activity**		
**Characteristics**			
	**n**	**mean ± SD**	**p-value**
**Indoor facilities**			0.348
No	1038	111.4 ± 19.8	
Yes	399	112.5 ± 19.9	
**School facilities**			<0.001
No	1058	110.5 ± 19.5	
Yes	379	114.9 ± 20.1	
**Playing fields**			0.017
No	725	110.5 ± 19.9	
Yes	712	112.9 ± 19.6	
**Bike/hiking/walking trails, paths**			0.044
No	701	110.6 ± 20.0	
Yes	736	112.7 ± 19.6	
**Public parks**			0.797
No	550	111.5 ± 19.5	
Yes	887	111.8 ± 20.0	
**Sex**			0.190
Male	697	112.4 ± 19.9	
Female	735	111.0 ± 19.7	
**Grade**			<0.001
Middle school or below	585	128.2 ± 11.9	
High school	846	100.4 ± 15.7	
**Race/ethnicity**			0.386
Hispanic	143	113.4 ± 19.0	
Black	232	112.2 ± 20.4	
White	920	111.2 ± 19.2	
Other	127	113.6 ± 23.0	
**School type**			0.615
Public	1223	111.6 ± 19.8	
Private	106	113.3 ± 18.5	
Other	106	110.8 ± 20.9	
**Work status**			<0.001
Do not work	1237	113.1 ± 19.7	
Work	197	102.8 ± 18.3	
**Crime, perceived neighborhood**			0.423
Strongly disagree	749	112.0 ± 18.8	
Somewhat disagree	343	110.5 ± 19.5	
Somewhat agree	211	111.6 ± 21.1	
Strongly agree	125	113.8 ± 23.5	
**Traffic, perceived neighborhood**			0.069
Strongly disagree	567	112.8 ± 19.5	
Somewhat disagree	378	109.5 ± 18.9	
Somewhat agree	335	111.9 ± 20.3	
Strongly agree	145	113. ± 21.4	
**Physical activity with friends**			<0.001
Disagree	257	100.8 ± 19.9	
Neutral	140	105.9 ± 18.8	
Agree	1034	115.2 ± 18.7	
**Friends’ physical activity, perceived**			<0.001
Disagree	293	104.7 ± 20.3	
Neutral	301	109.6 ± 19.2	
Agree	817	115.0 ± 19.1	
**Family physical inactivity**			<0.001
Disagree	949	113.3 ± 19.4	
Neutral	252	107.8 ± 20.8	
Agree	231	109.2 ± 19.3	

### 2.4 Ethical considerations

All data accessed were fully anonymized and without any of the 18 direct identifiers according to the Health Insurance Portability and Accountability Act. Ethical approval was waived by the Florida International University Health Sciences IRB since the analysis was considered non-human subjects research using de-identified data.

## 3 Results

Adolescents that reported availability of school recreational facilities had a statistically significantly higher mean daily MVPA (114.9 min) as compared with those without availability (110.5 min; p < 0.001; Table 1). Adolescents with availability of playing fields had a statistically significantly higher mean of daily MVPA (112.9 min) compared with those who did not (110.5 min daily; p = 0.017). Also, those with access to bike, hiking or walking trails had a higher mean MVPA (112.7 min) than those who did not (110.6 min; p = 0.044). There was no significant difference in mean daily minutes MVPA for availability of indoor facilities. Mean daily minutes of MVPA was different between adolescents in middle school or below (128.2 min) compared with high schoolers (100.4 min daily; p < 0.001). Mean daily minutes of MVPA was different for those whose parents worked compared with those who did not work (102.8 min vs. 113.1 min, p value < 0.001). Moreover, mean daily minutes of MVPA were different amongst respondents based upon whether they practice physical activity with friends (115.2 min for agree vs 105.9 min for neutral vs 100.8 min for disagree, p < 0.001). Mean daily minutes of MVPA was different amongst respondents based upon whether their friends were physically active or not (115.0 min for agree vs 109.6 for neutral vs 104.7 for disagree, p < 0.001). No statistically significant differences in mean daily minutes of MVPA were seen for sex, race/ethnicity, school type, perceived neighborhood crime, or perceived neighborhood traffic.

In testing the associations between the type of recreational facilities and the average daily minutes of MVPA of US adolescents (Table 2), the unadjusted models revealed that availability of school recreational facilities and playing fields were positively associated with an additional 4.4 min per day (95 % CI 2.1, 6.7) and 2.5 min per day (95 % CI 0.4, 4.5) of MVPA of adolescents, respectively. No statistically significant associations between MVPA and indoor recreational facilities availability, public parks or bike/hiking/walking trains or paths were found. In fully adjusted models, the average daily minutes of MVPA differed by 4.4 min (95 % CI 2.6, 6.2) if participants had school recreational facilities. No positive association with MVPA was found for having indoor recreational facilities in the adjusted model. Furthermore, no statistically significant associations between playing field or bike/hiking/walking trains or paths and MVPA and were found. However, the average daily minutes of MVPA was 1.8 min lower (95 % CI 2.6, 6.2) if participants had access to public parks when controlling for the covariates. After adjustment, other characteristics exhibited statistically significant associations with average daily minutes of MVPA including PA with friends (an additional 8.9 min daily; 95 % CI 6.7, 11.1) and disagreeing with family physical inactivity status (an additional 2.6 min daily; 95 % CI 0.6, 4.6). In addition, the adjusted model showed statistically significant inverse associations between high school status as well as working status with average daily minutes of MVPA. High school students engaged in 26.7 fewer minutes of MVPA daily (95 % CI −28.2, −25.2) than middle school students. Parental work status was also associated with an average difference of 2.4 min MVPA daily (95 % CI −4.6, −0.2) compared with those whose parents did not work.

**Table 2:** Unadjusted and Adjusted associations between type of recreational facility and Moderate to Vigorous Physical Activity in 2017.

**Characteristics**	**Unadjusted**	**Adjusted**
	**Minutes (95% CI^1^)**	**Minutes (95% CI)**
**Indoor facilities**		
No	Ref^2^	ref
Yes	1.1 (-1.2, 3.4)	0.4 (-1.4, 2.1)
**School facilities**		
No	ref	ref
Yes	4.4 (2.1, 6.7)	4.4 (2.6, 6.2)
**Playing fields**		
No	Ref.	Ref.
Yes	2.5 (0.4, 4.5)	1.7 (-0.02, 3.43)
**Bike/hiking/walking trails, paths**		
No	Ref.	Ref.
Yes	1.7 (-0.6, 4.1)	0.64 (-0.9, 2.2)
**Public parks**		
No	Ref.	Ref.
Yes	0.3 (-1.8, 2.4)	−1.8 (-3.4, −0.1)
**Sex**		
Male	ref	ref
Female	−1.4 (-3.4, 0.7)	−0.3 (-1.7, 1,1)
**Age**	−10.7 (-11.0, −10.3)	NA
**Grade**		
Middle school or below	ref	ref
High school	−27.9 (-29.4, −26.3)	−26.7 (-28.2, −25.2)
**Race/ethnicity**		
White	ref	ref
Black	1.0 (-1.9, 3.8)	−0.7 (-2.7, 1.3)
Hispanic	2.2 (-1.2, 5.7)	1.1 (-1.3, 3.5)
Other	2.5 (-1.2, 6.1)	0.1 (-2.4, 2.7)
**School type**		
Public	ref	ref
Private	1.7 (-2.2, 5.7)	1.9 (-0.8, 4.7)
Other	−0.8 (-4.7, 3.1)	−0.9 (-3.6, 1.9)
**Parent Work status**		
Do not work	ref	ref
Work	−10.2 (-13.2, −7.3)	−2.4 (-4.5, −0.2)
**Crime, perceived neighborhood**		
Strongly disagree	ref	ref
Somewhat disagree	−1.5 (-4.0, 1.0)	−0.4 (-2.2, 1.5)
Somewhat agree	−0.4 (-3.3, 2.7)	0.5 (-1.8, 2.7)
Strongly agree	1.8 (−2.0, 5.5)	2.1 (-0.7, 4.8)
**Traffic, perceived neighborhood**		
Strongly disagree	ref	ref
Somewhat disagree	−3.3 (-5.8, −0.7)	−0.9 (-2.8, 1.0)
Somewhat agree	−0.9 (-3.6, 1.8)	−0.2 (-2.1, 1.8)
Strongly agree	0.35 (-3.3, 4.0)	0.1 (-2.6, 2.8)
**Physical activity with friends**		
Disagree	ref	ref
Neutral	5.1 (1.2, 9.0)	2.0 (-1.0, 4.9)
Agree	14.4 (11.8, 17.0)	8.9 (6.7, 11.1)
**Friends’ physical activity, perceived**		
Disagree	ref	ref
Neutral	4.9 (1.8, 8.0)	−1.2 (-3.6, 1.1)
Agree	10.2 (7.7, 12.8)	1.2 (-1.0, 3.3)
**Family physical inactivity**		
Agree	ref	ref
Disagree	4.1 (1.3, 6.9)	2.6 (0.6, 4.6)
Neutral	−1.3 (-4.8, 2.2)	−1.0 (-3.5, 1.4)

## 4 Discussion

This study assessed the availability of recreational facilities and average minutes of moderate to vigorous physical activity (MVPA) per day of US adolescents in the FLASHE cohort. Adjusted models showed that among all recreational facilities studied, those in school settings exhibited significant positive associations with MVPA of adolescents whereas access to public parks was negatively associated with MVPA.

Regarding physical environment and PA association among adolescents, a recent systematic review suggested that the most frequent settings assessed were the outdoor neighborhood, indoors, and school environments (Kelso et al., 2021), with most research focused on the outdoor neighborhood (Kowaleski-Jones et al., 2016, McGrath et al., 2015). Findings related to covariates such as sociodemographic characteristics that resulted in significant associations with MVPA corroborated those in previous studies (Wyszyńska et al., 2020, Brooke et al., 2016, Hnatiuk et al., 2019). After adjusting for these covariates, the statistically significant direct association between availability of school recreational facilities and MVPA of adolescents remained. This finding may be explained by schools representing a primary setting where adolescents spend most of their time and where significant opportunities to increase PA may be encouraged (Prince et al., 2019). Implementation of physical education (PE) programs, accessibility to play facilities and equipment, and availability of supervised after-school fields have been reported to be positively associated with higher PA among adolescents (Wyszyńska et al., 2020, Durant et al., 2009). Additionally, an analysis found that more than half of middle and high school students participated in school sports and most middle schools and high schools provided interscholastic sports teams (Pate et al., 2006).

In support of the findings, a systematic review reported that the school environment can account for up to 37.6 min of MVPA daily, with a higher number of minutes in outdoor school settings (Kelso et al., 2021). The same review also reported that schoolyards were the settings where the lowest amount of sedentary time was spent by adolescents (Kelso et al., 2021). When considering the timing of PA at school recreational facilities, studies have reported the main contributor to MVPA is afterschool time, especially PA that is supervised (Thornton et al., 2017, Beets et al., 2009, Sallis et al., 2001). However, the overall features of school recreational facilities rather than individual characteristics may be more important for achieving MVPA (Nichol et al., 2009).

Access to certain outdoor facilities such as playing fields, public parks and trails have been shown to exhibit a positive association with PA among adolescents (Kowaleski-Jones et al., 2016, McGrath et al., 2015, Prince et al., 2019, McCrorie et al., 2014). Two different literature reviews reported that outdoor environments were one of the most important settings when accounting for total minutes of MVPA (Kelso et al., 2021, Prince et al., 2019). These findings are expected as roads, streets, and transportation environments have been reported to importantly contribute to MVPA among adolescents (Prince et al., 2019, McCrorie et al., 2014). The association between the transportation environment and higher PA could be explained by different features that may enhance active transportation to school or other locations within the neighborhood (Lanza et al., 2020). Despite this, results from the present study do not corroborate these findings. Even more, access to public parks where negatively associated with MVPA in our study. In line with this finding, parks and greenspaces have accounted for a relatively low amount of MVPA in youth when analyzed separately in a previous study (McCrorie et al., 2014). A study examining adolescents’ PA motivations noted that neighborhood parks are often designed for children, rather than for adolescents who prefer sports facilities such as basketball courts, tennis courts, swimming pools, and tracks (Ries et al., 2008).

The relation between indoor facilities and PA of adolescents has previously been mixed, where some studies suggested that indoor environments mainly account for light PA and sedentary time rather than MVPA (Prince et al., 2019), and others report that indoor recreation facilities are associated with increases in vigorous PA levels (Niclasen et al., 2012) and higher minutes of MVPA levels (Kelso et al., 2021). Furthermore, specific indoor facilities such as home environments have suggested mixed results with adolescent MVPA (Carlson et al., 2016, Klinker et al., 2014). The results of the present study align with the evidence suggesting indoor environments may not be a core contributor to significant amounts of MVPA among US adolescents.

Finally, shared use agreements between schools and the community may be explored to promote more PA at school facilities. Educational institutions often have both indoor and outdoor facilities that are not in use outside of school hours. Through shared use agreements, schools may work in partnership with municipalities to allow the public to have access to safe recreational facilities to improve their PA levels, health, and well-being (Spengler et al., 2010, Kanters et al., 2014, Chace and Vilvens, 2015). According to the 2014 School Health Policies and Practice Study, 46 % of the schools in the US had signed agreements for the use of their recreational facilities by the public outside of school hours (Cdc, 2014). Spengler et al, on the other hand, revealed that seven out of ten schools in 48 states reported to have agreements to share school recreational facilities with the community (Spengler et al., 2010).

Our study has limitations worth noting. Since the focus of the study was on setting rather than timing, the moments at which PA occurred within the school were not assessed making it impossible to differentiate between minutes of MVPA accumulated before, during, or after school hours. Previous studies have suggested that there are differences in timing of PA during school (Kelso et al., 2021, Thornton et al., 2017, Prince et al., 2019, Beets et al., 2009, Sallis et al., 2001), for which caution when interpreting the current results is suggested. Nevertheless, school recreational facilities remain to be a central setting for accruing minutes of MVPA among US adolescents. Additionally, the current study assessed the availability of different recreational facilities rather than access or actual use of the settings. Since these two terms represent different features of physical environments, caution should be used when interpreting the results.

## 5 Conclusion

The findings herein bring focus to the importance of school recreational facilities for MVPA of adolescents in the US. In 2014, less than half of US schools opened their recreational facilities for public use outside of school hours, and only about 9–34.3 % of US schools required physical education programs from sixth to twelfth grade (Department of Health and Human Services, 2014). Our finding that having school recreational facilities was associated with 4.7 more minutes of MVPA could be considered significant as previous research has shown that adolescents tend to accumulate around 66 %, 16 %, and 18 % of their PA in sporadic bouts lasting 1–4 min, 5–9 min, and more than 10 min, respectively (Mark and Janssen, 2009). Moreover, evidence suggests that as youth age, they tend to increase the frequency rather than the duration of bouts of MVPA (Brooke et al., 2016, Hnatiuk et al., 2019), and the magnitude found in the current study comports with previous work (Brooke et al., 2016). Ultimately, findings from this study support the need for focused studies and specific policies that address school recreational facilities for increasing MVPA among adolescents.

## Conflict of interest

None declared.

## CRediT authorship contribution statement

**Jamee Guerra:** Writing – review & editing, Writing – original draft, Validation, Methodology, Investigation, Formal analysis. **Johnatan Jhon:** Writing – review & editing, Methodology, Investigation, Formal analysis, Conceptualization. **Kevin Lanza:** Writing – review & editing, Validation, Supervision, Methodology, Conceptualization. **Grettel Castro:** Writing – review & editing, Supervision, Software, Methodology, Formal analysis, Data curation, Conceptualization. **Noël C. Barengo:** .

## Declaration of competing interest

The authors declare that they have no known competing financial interests or personal relationships that could have appeared to influence the work reported in this paper.

## Data Availability

Data will be made available on request.
